# Food Handling Practices Among Food Businesses in Jigjiga, Eastern Ethiopia, During the COVID-19 Pandemic: Cross-Sectional Study

**DOI:** 10.2196/64214

**Published:** 2025-08-20

**Authors:** Alinoor Mohamed Farah, Tahir Yousuf Nour, Abdurahman Kedir Roble, Muse Obsiye, Mowlid Akil Aden, Abdulahi Siraj Abdi, Fentabil Getnet

**Affiliations:** 1 School of Public, College of Medicine and Health Sciences, Jigjiga University, CoMHS Building, 2nd Floor, Jigjiga, 1020, Ethiopia, 251 911053913; 2 School of Nursing and Midwifery, College of Medicine and Health Sciences, Jigjiga University, Jigjiga, Ethiopia

**Keywords:** COVID-19, knowledge, practice, Ethiopia, food safety, food, food supply, good hygiene practices, proper hygiene, hygiene, cross-sectional study, data collection, employee, food handler, food safety training, food handling

## Abstract

**Background:**

The COVID-19 pandemic has posed significant challenges to food safety practices globally, profoundly affecting the knowledge, attitudes, and practices of both food handlers and consumers.

**Objective:**

This study aimed to investigate food safety knowledge and practices of food handlers in the context of COVID-19.

**Methods:**

A cross-sectional study was conducted in Jigjiga during the pandemic. A total of 384 food handlers were surveyed using a structured questionnaire and an observational checklist. The questionnaire assessed knowledge of COVID-19 symptoms, transmission, and prevention measures, and the checklist evaluated food safety practices and the implementation of COVID-19 prevention measures in food businesses. Categorical variables (eg, sufficient vs insufficient COVID-19 knowledge and good vs poor food-safety practice) were summarized as frequencies and percentages. Pearson chi‐square test was used to assess differences in these binary outcomes across demographic and other categorical subgroups (eg, sex, age category, education level, and source of COVID-19 information). A *P* value <.05 was considered statistically significant.

**Results:**

A total of 384 food handlers were approached, and all responded (response rate=100%). The majority of participants (276/384, 71.9%) had received food hygiene training, and the main source of COVID-19 information was government news media (170/384, 44.3%). The majority of respondents (264/384, 68.8%) correctly identified the key COVID‑19 symptoms, and 52.1% (200/384) accurately understood that respiratory droplets from coughs or sneezes drive transmission. However, less than 50% of participants consistently practiced preventive measures such as avoiding handshaking, frequently sanitizing food contact surfaces, and reminding customers to follow physical distancing. Participants who obtained information from government sites and the media had sufficient knowledge compared to other participants (*P*=.07). Females (*P*=.03), younger adults (*P*=.03), married individuals (*P*=.04), those with secondary education (*P*=.014), and those who had received previous food safety training (*P*=.004) demonstrated better food handling practices than their counterparts. Furthermore, 61.4% (236/384) of food businesses had handwashing facilities at the entrance, 70.2% (270/384) implemented crowd control measures, and 56.1% (215/384) used floor markings to facilitate physical distancing. However, only 57.7% (221/384) of food establishments routinely cleaned and disinfected their work surfaces and touch points.

**Conclusions:**

These findings highlight the need for targeted education and training interventions to improve food handlers' knowledge and practices, particularly during the COVID-19 pandemic.

## Introduction

The COVID-19 pandemic significantly impacted food safety practices and consumer behavior. Although there is no evidence of SARS-CoV-2 transmission through food [1,2], the virus can survive on surfaces, including food packaging [3]. Consequently, food handlers and consumers have adopted enhanced hygiene measures, such as increased handwashing, produce cleaning, and food thermometer use [4,5]. The food industry has implemented additional practices to protect workers, including social distancing, face coverings, and employee screening [6]. However, pandemic-related disruptions may increase food safety risks [6]. To mitigate these risks, experts recommend adhering to established food safety protocols, proper sanitization, and maintaining good hygiene practices throughout the food supply chain [7]. Postpandemic, it is expected that there will be a greater understanding of the importance of food hygiene and creative adaptations in food service [2].

Food handlers in businesses demonstrated improved knowledge and attitudes toward food safety during the pandemic [8]. Although home-based online food businesses have expanded, food handlers have shown low knowledge and improper practices in some areas [9]. Age, income, education, and gender influence food safety practices [4]. Overall, the pandemic increased awareness of food safety but also led to some misconceptions and improper practices, highlighting the need for targeted educational programs [5,10].

Due to numerous factors, food safety is significantly more complicated in developing nations. Poverty is one of the leading causes of unsafe food consumption due to a lack of access to sufficient food and clean water, poor government structure, the persistence of infectious diseases in the community, inconvenient environmental conditions to ensure food safety, and poor food handling and sanitation practices [11,12]. Ethiopia is not exceptional because of the prevalence of poor food handling and sanitation practices, inadequate food safety laws, weak regulatory systems, lack of financial resources to invest in food safety, and lack of training and education for food handlers [12].

In this context, safeguarding the food supply chain during the COVID-19 pandemic requires not only industry-level interventions but also strong frontline food safety practices at every point of contact. In Ethiopia, where food handling often occurs under resource constraints and regulatory oversight may be limited, understanding how food handlers adapt and where gaps remain is critically important. Although several studies have documented shifts in knowledge and attitudes among food handlers, few have examined these dynamics in resource-constrained settings, where unique socioeconomic and infrastructural challenges may amplify both COVID-19–related and other food safety risks. Therefore, this study aimed to investigate the food safety knowledge and practices of food handlers in Jigjiga Town. The findings will inform targeted, context-appropriate training and policy measures to reinforce safe food handling practices both during and after the COVID-19 era.

## Methods

### Study Design, Setting, and Period

A cross-sectional study was conducted from August 12 to 23, 2020, to evaluate the food safety knowledge and practices of food handlers and assess the sanitary conditions of groceries in Jigjiga town. Jigjiga is the capital town of the Somali Regional State located in the eastern part of Ethiopia, and the city has an elevation of 1609 meters above sea level. Based on the 2007 Census conducted by the Central Statistical Agency of Ethiopia, Jijiga had a total population of 203,588, of whom 109,138 were men and 94,450 were women. The town is divided into 30 kebeles (the smallest administrative units), of which 20 are urban and 10 are rural ones.

### Sample Size Determination

The sample size was determined using Epi Info version 3.5.1 software (Center for Disease Control and Prevention, Atlanta, 2004) by single population proportion formula with the assumptions of 95% confidence level and 5% precision. A sample size of 384 was estimated with the conservative assumption of a 50% prevalence of good food safety practices, with 95% certainty.

### Sampling Technique

A master list of 519 food-vending outlets, including shops, grocery stores, supermarkets, and minimarkets, was obtained from the Jigjiga City Council Office. To capture unregistered businesses, we conducted a door-to-door census in each kebele, which identified an additional 201 outlets and increased the total sampling frame to 720 establishments. A proportional sample size was determined for each kebele, and groceries were randomly selected from each kebele using a random-number generator. A total of 384 grocery stores and shops were randomly selected for the study. The study targeted both food retail establishments (grocery stores, supermarkets, minimarkets, and food shops) and the individual food handlers working within them.

### Inclusion and Exclusion Criteria

Food vending outlets, specifically shops, grocery stores, supermarkets, and mini-markets, regardless of whether they were listed or registered by the Jigjiga City Council office, were included.

### Data Collection and Quality Control

In total, 4 sanitarians were recruited as data collectors and supervisors for the study. All team members participated in an orientation covering the study objectives, questionnaire components, and data quality management. We used a structured questionnaire that was pretested on 10% (38/384) of a similar study population, and expert feedback was incorporated into the final tool.

The questionnaire was drafted in English and translated into Somali using forward–backward translation to ensure semantic equivalence of the questions. It included items on common COVID-19 symptoms and food handling knowledge, adapted from the World Health Organization (WHO) and Food and Agriculture Organization (FAO) COVID-19 and food safety protocols [13]. Data collectors interviewed food handlers in stores, shops, and groceries and observed their on-the-job practices using a standardized checklist to assess prevention measures.

We evaluated the internal consistency of the tools using Cronbach α (α=0.702), indicating acceptable reliability. For observational data, we conducted interrater reliability exercises: data collectors applied the checklist side by side, discrepancies were discussed, and retraining was provided until the standardized criteria were consistently applied. The final questionnaire was refined to improve clarity, minimize bias, and align with the study objectives.

We took multiple precautions to ensure that the participants did not feel compelled to provide socially desirable responses. First, the questionnaire was carefully worded and structured so that the questions were neutral and nonleading. Before data collection began, the participants were assured that their answers would remain anonymous, used only in aggregate form, and would have no impact on their employment or licensing status. This reassurance helped reduce the fear of negative consequences for honest reporting. The interviewers received dedicated training in neutral probing techniques and were instructed to avoid any verbal or nonverbal cues that might suggest approval or disapproval of particular answers. Finally, the participants were reminded that their honest feedback was valuable and that they could pause or take brief breaks at any time, helping ensure that the respondents remained attentive and invested in providing thoughtful answers.

### Methods of Data Analysis

Data were coded and entered into Epi Info version 3.5.1 software and exported into STATA version 14 for analysis. Knowledge and practice questions were scored as 1 or 0 for correct and incorrect responses, respectively. Mean scores were calculated and used as cut-off points to dichotomize the outcome variables. Scores below the mean score were considered as indicating inadequate knowledge or practice, whereas scores equal to or above the mean score were considered as indicating adequate knowledge and favorable adequate practice. Knowledge questions were scored as 1 or 0 for correct and incorrect responses, respectively. The total knowledge score ranged from 0 (no correct answers) to 6 (all correct answers); a mean score of ≤3.5 indicated poor knowledge, and a mean score of >3.5 indicated good knowledge. There were 14 practice questions scored on a 3-point scale from 1 (“always”) to 3 (“never”). Total practice scores ranged from 14 to 42; a mean score of >18.8 (answering “never” or “occasionally”) indicated poor practices, and a score of ≤18.8 (answering “always”) indicated good practices. Thus, the lower the practice scores, the higher the probability of good practices, and vice versa.

Summary statistics, such as frequencies and proportions, were computed as appropriate. Categorical variables (eg, sufficient vs insufficient COVID-19 knowledge and good vs poor food-safety practice) were summarized as frequencies and percentages. Pearson’s chi‐square test was used to assess differences in these binary outcomes across demographic and other categorical subgroups (eg, sex, age category, education level, and source of COVID-19 information). *P* value <.05 was considered statistically significant.

### Operation Definition

Food handling refers to one or more operations of food production, manufacture, offering or displaying for sale, storage, preservation, wrapping, transportation, delivery, importation, exportation, or the licensing or approval for any of such activities.

Food handling knowledge refers to a food handler’s accurate understanding of COVID-19–related health and safety information as it applies to one or more operations of food production, manufacture, sale, storage, preservation, wrapping, transportation, delivery, importation, exportation, or licensing or approval of such activities.

Food handling practices refer to the consistency with which food handlers perform COVID-19–related safety measures, such as personal protective equipment use, hand hygiene, respiratory etiquette, surface sanitation, and physical distancing, across all stages of food production, sale, storage, transport, or regulatory activities.

COVID-19 prevention measures refer to the extent to which a food retail establishment implements structural and managerial interventions designed to reduce viral transmission.

Food businesses or establishments refer to any commercial establishment engaged in the retail sale of edible products, including but not limited to supermarkets, grocery stores, and convenience shops.

### Ethical Considerations

This study was conducted in accordance with the guidelines of the Declaration of Helsinki, and all procedures involving research participants were approved by the Jigjiga University institutional review board (RERC/039/2020). Written informed consent was obtained from all participants. The participants were not compensated for participating in the study. The study participants’ data were confidential and protected.

## Results

### Sociodemographic Characteristics

A total of 384 food handlers were approached, and all of them responded (response rate=100%). As shown in Table 1, of the total participants, 50.8% were male, with a mean age of 32.1 (SD 4.8) years. Out of 384, the majority of participants were under 40 years of age (n=369, 96.1%) and 108 (28.1%) respondents had college and university-level education, and 296 (77.1%) were married. The majority of the participants received food hygiene training (n=276, 71.9%), and 108 (28.1%) were working at grocery stores. The main source of information about COVID-19 among participants was government news media (n=170, 44.3%).

**Table 1.** Sociodemographic characteristics of food handlers in food businesses in Jigjiga, Eastern Ethiopia (2020).

Variable	Value
Sex, n (%)	
Male	195 (50.8)
Female	189 (49.2)
Age (years), mean (SD)	32.1 (4.8)
18‐39	369 (96.1)
>40	15 (3.9)
Marital status, n (%)	
Married	296 (77.1)
Single	57 (14.8)
Divorced	24 (6.3)
Widowed	7 (1.8)
Education status, n (%)	
No education	98 (25.5)
Elementary	82 (21.4)
Secondary	96 (25.0)
College and university	108 (28.1)
Type of food business, n (%)	
Wholesale shops	104 (27.1)
Retail shops	95 (24.7)
Grocery store	108 (28.1)
Minimarket/supermarket	77 (20.1)
License status, n (%)	
Licensed	249 (64.8)
Not licensed	135 (35.2)
Food hygiene training, n (%)	
Yes	276 (71.9)
No	108 (28.1)
Source of information on COVID-19, n (%)	
Government news media	170 (44.3)
Social media	143 (37.2)
Other	71 (18.5)

### Knowledge

As shown in Table 2, the majority of respondents (264/384, 68.8%) correctly identified the key COVID‑19 symptoms. The majority of the respondents understood the dynamics of COVID-19 infectiousness: 63.8% (245/384) of respondents were aware of the possibility of infection before the onset of symptoms; 52.1% (200/384) of the participants responded true on droplets as a major transmission route. More than half of the respondents (215/384, 56.0%) understood the need for self-isolation, and 54.4% (209/384) were aware of the standard precautions that need to be followed when dealing with customers.

**Table 2.** Knowledge of food handlers in food businesses about COVID-19 transmission, prevention, and food safety practices in Jigjiga, Eastern Ethiopia (2020).

Questions	Response (n=384)		
Knowledge	True, n (%)	False, n (%)	I do not know, n (%)
The common symptoms of COVID-19 are fever, fatigue, and dry cough.	264 (68.8)	93 (24.2)	27 (7.0)
COVID-19 can be transmitted through direct contact of respiratory droplets when infected persons cough or sneeze.	200 (52.1)	156 (40.6)	28 (7.3)
The disease can be transmitted from asymptomatic patients.	245 (63.8)	110 (28.7)	29 (7.5)
The disease can be spread by touching a surface or object that has the virus on it and then touching their own mouth, nose, or possibly their eyes.	214 (55.7)	135 (35.2)	35 (9.1)
A person with mild symptoms of COVID-19 must remain at home until resolution of clinical symptoms.	215 (56.0)	126 (32.8)	43 (11.2)
Standard precautions such as wearing gloves and masks should be followed by food handlers when dealing with customers.	209 (54.4)	111 (28.9)	64 (16.7)

### Practice

Table 3 explains the practical measures in place by the food handlers to protect themselves and their customers; 52.9% (203/384) of the respondents reported always wearing masks and gloves when dealing with the customers, and up to 51.3% (197/384) washed their hands when they were soiled. Other measures included maintaining social distance and avoiding shaking hands, hugging, or kissing. Unfortunately, less than 50% (192/384) of the participants had avoided shaking hands, touching their nose and mouth, frequently sanitized all food contact surfaces, and made regular announcements to remind their customers to follow physical distancing.

**Table 3.** Food safety and hygiene practices of food handlers in food businesses in Jigjiga, Eastern Ethiopia (2020).

Questions	Response (n=384)		
Practice	Always, n (%)	Occasionally, n (%)	Never, n (%)
I always wear masks and gloves when dealing with customers.	203 (52.9)	136 (35.4)	45 (11.7)
I wash my hands with water and soap before putting on the gloves.	176 (45.8)	191 (49.8)	17 (4.4)
I use sanitizer between customers (before and after handling food/packing food).	202 (52.6)	104 (27.1)	78 (20.3)
I wash my hands whenever they are soiled.	197 (51.3)	104 (27.1)	83 (21.6)
I wash my hands after blowing my nose or covering a sneeze.	188 (49.0)	113 (29.4)	83 (21.6)
When I’m sneezing or coughing, I cover my nose or mouth with a tissue/clean cloth or into my elbow.	146 (38.0)	122 (31.8)	116 (30.2)
I keep at least one-meter distance from my work colleagues and the customers.	217 (56.5)	96 (25.0)	71 (18.5)
I avoid shaking hands, hugging, or kissing with colleagues/customers.	184 (47.9)	127 (33.1)	73 (19.0)
I avoid touching my mouth, nose, and eyes with my hand if I have not washed them with water and soap.	183 (47.7)	123 (32.0)	78 (20.3)
I frequently wash and sanitize all food contact surfaces.	178 (46.4)	128 (33.3)	78 (20.3)
I make regular announcements to remind customers to follow physical distancing advice and clean their hands regularly.	142 (37.0)	121 (31.5)	121 (31.5)

### COVID-19 Prevention Measures

As shown in Table 4, 61.4% (235/383) of food businesses had handwashing facilities at the entrance. Furthermore, 70.2% (269/384) of food businesses were able to control the crowd. More than half (215/383, 56.1%) of the food businesses used floor markings inside the retail store to facilitate compliance with physical distancing. Moreover, 74.7% (286/383) and 54.1% (207/383) of the food businesses had barriers at tills and counters introduced as an additional level of protection for food handlers and signs placed at entry points to request customers not to enter the shop if they were unwell or had COVID-19 symptoms, respectively. In addition, 57.7% (221/383) of food businesses frequently cleaned or disinfected work surfaces and touch points.

**Table 4.** COVID-19 prevention measures practiced by food handlers in food businesses in Jigjiga, Eastern Ethiopia (2020).

COVID-19 prevention measures	Response, (n=383)	
	Yes, n (%)	No, n (%)
Handwashing facility observed at the establishment.	235 (61.4)	148 (38.6)
Queue/crowd control managed.	269 (70.2)	114 (29.8)
Floor markings used inside the retail store to facilitate compliance with the physical distancing, particularly in the most crowded areas, such as serving counters and tills.	215 (56.1)	168 (43.9)
Barriers at tills and counters introduced as an additional level of protection for food handler.	286 (74.7)	97 (25.3)
Signs placed at entry points to request customers not to enter the shop if they are unwell or have COVID-19 symptoms.	207 (54.1)	176 (45.9)
Frequent cleaning/disinfection of work surfaces and touch points.	221 (57.7)	162 (42.3)

### Comparison of COVID-19 Knowledge and Practice Between Different Groups

As shown in Table 5, less than half (191/384, 49.7%) of the respondents had sufficient knowledge. Of the total participants, more male participants had sufficient knowledge than female participants (n=100, 52.4% vs n=91, 47.6%), and the difference was not statistically significant (*P*=.54). More participants who obtained information from government sites and the media had sufficient knowledge than other participants (*P*=.07). Female (*P*=.03), younger (*P*=.06), and married respondents (*P*=.04) practiced more than their counterparts. Furthermore, respondents with secondary education (*P*=.01) and those who received food hygiene training (*P*=.004) had better practice than their counterparts.

**Table 5.** Comparison of COVID-19 knowledge and food safety practices among food handlers across sociodemographic groups in Jigjiga, Eastern Ethiopia (2020).

Variable	Sufficient knowledge (n=384)		Good practice (n=384)	
	Frequency, n (%)	*P* value^a^	Frequency, n (%)	*P* value^a^
Overall	191 (49.7)		233 (60.7)	
Sex		.54		.03^b^
Male	100 (52.4)		108 (46.4)	
Female	91 (47.6)		125 (53.6)	
Age (years)		.78		.006^b^
18‐39	183 (95.8)		229 (98.3)	
>40	8 (4.2)		4 (1.7)	
Marital status		.52		.04^b^
Married	145 (75.9)		189 (81.1)	
Single	32 (16.7)		32 (13.7)	
Divorced	12 (6.4)		9 (3.9)	
Widowed	2 (1.0)		3 (1.3)	
Education status		.35		.01^b^
No education	51 (26.7)		60 (25.7)	
Elementary	37 (19.4)		38 (16.3)	
Secondary	43 (22.5)		67 (28.8)	
College and university	60 (31.4)		68 (29.2)	
Type of food business		.56		.35
Wholesale	52 (27.2)		61(26.2)	
Retail	47 (24.6)		53(22.8)	
Grocery	49 (25.7)		73(31.3)	
Minimarket/supermarket	43 (22.5)		46(19.7)	
Food hygiene training		.77		.004^b^
Yes	136/191 (71.2)		180 (77.3)	
No	55/191 (28.8)		53 (22.7)	
License status		.38		.37
Licensed	128 (67.0)		147 (63.1)	
Not licensed	63 (33.0)		86 (36.9)	
Source of information on COVID-19		.07		.52
Government site and media	94 (49.2)		98 (42.1)	
Social media	69 (36.1)		89 (38.2)	
Other	28 (14.7)		46 (19.7)	

a Pearson chi-squared test to compare knowledge, attitudes, and practices between different groups.

b Statistically significant at *P*<.05

## Discussion

### Principal Findings

This study examined food safety knowledge and practices among 384 food handlers in Jigjiga Town during the COVID-19 outbreak. More than half of the participants were not adequately informed about COVID-19. Higher levels of COVID-19 knowledge were observed among men, older individuals, and those with a college or university education. Studies conducted in different countries, including India, Jordan, Malaysia, and Bangladesh, have shown that food handlers generally possess fair to excellent knowledge, positive attitudes, and good practices regarding food safety during the pandemic [8,11,12,14]. However, some studies have identified areas for improvement, particularly in cross-contamination prevention and safe food handling practices [9].

The food safety practices of the female participants were better than those of the male participants, which is consistent with the findings of other studies [15,16]. This could be explained by the fact that females are more involved in-home food preparation, multitask more, and pay more attention to COVID-19. Similarly, younger respondents and married respondents practiced more than their counterparts. Our findings disagree with those of other studies that have shown insufficient practice among younger adults [17].

Participants with secondary education and those trained in food safety had better practice than their counterparts, which is in line with other similar studies conducted in Iran and Malaysia [14,16]. Attending food safety courses was also found to be a significant factor influencing food safety knowledge and practices among Malaysian food truck vendors [14]. A brief educational intervention study on restaurant food handlers’ knowledge of food allergies conducted in Brighton in 2014 revealed improvements in absolute knowledge and practice change [18]. Food safety and hygiene interventions influenced the food safety knowledge, behavior, and habits of employees in the food and beverage departments of hotels and restaurants in Turkey’s tourism industry [19]. A systematic review conducted in Canada found that routine intervention education of food service premises is effective in reducing the risk of foodborne illnesses and improving food handler knowledge and practices. Community-based education programs have been shown to increase public awareness of food safety [20].

Participants who obtained information from government websites and traditional media had higher levels of knowledge. In our study, most respondents relied on government media for information updates. Although government agencies, healthcare professionals, and scientists are generally trusted sources of food safety information, social media is considered less reliable [21]. However, the primary information source can significantly influence the link between COVID-19 knowledge and protective behaviors; interpersonal communication and social media often elicit stronger behavior change than official channels [22].

The food service industry was among the first frontline employment sectors to experience the effects of the COVID-19 pandemic. To date, no study has indicated that COVID-19 spreads through food. In addition, there is no evidence that viruses that infect the respiratory tract can be transmitted through food or food packaging [7]. SARS-CoV and Middle East Respiratory Syndrome Coronavirus do not appear to be transmitted through food consumption [7].

According to the FAO and World Food Program, touching food packages or containers contaminated with SARS-CoV-2 could transmit the virus to the mouth, nose, or eyes [7]. However, because the virus does not survive on these surfaces, this is not considered the primary route of disease transmission. A previous study found that food products can be used to spread respiratory viruses, such as SARS-CoV-1 and influenza [23]. Another study found that the risk of Ebola infection in individual humans in the United States was negligible to low as a result of contaminated cocoa beans, palm oil, or cashews imported from South Africa [24].

More work is needed to prevent SARS-CoV-2 transmission from the respiratory tract to food packaging surfaces or through food consumption. The Food and Drug Administration has proposed guidelines for consumers to follow when shopping for, handling, and preparing food. To avoid SARS-CoV-2 transmission and comply with the food safety system’s minimum requirements, food handlers, including food establishment employees and consumers, should follow good sanitation and hygiene practice guidelines [25].

Ensuring smooth food movement through the supply chain requires vigilance and cooperation from all involved. It is essential to maintain consumer confidence in both food safety and availability by providing a secure environment at every stage, especially in retail shops and canteens, where both consumers and retailers must uphold rigorous hygiene and safety standards [7]. Retailers can help by providing sanitary facilities, such as wipes, disinfectants, and sanitizers, as well as displaying sanitary practices with visual aids. Physical separation can be maintained by marking the floors as a reference for the minimum distance. To avoid contact at cashier counters, plexiglass can be installed, and food tasting for promotional campaigns should be avoided during the pandemic [7].

Consumers should ensure that family members in vulnerable groups (immune-compromised, elderly, children, and COVID-19 patients) remain at home [26]. Furthermore, wearing masks, gloves, using hand sanitizers and wipes before handling food carts, avoiding reusable shopping bags, and practicing acceptable respiratory etiquette should be prioritized. If reusable bags are deemed regionally acceptable, they should be disinfected immediately after use [27].

This study had some limitations. First, its cross-sectional design precludes any causal inferences regarding the relationship between food handlers’ COVID-19 knowledge and their practices. Because the data were collected at a single point in time, we cannot determine whether greater knowledge drives better practices or vice versa, nor can we track how these measures evolve alongside changing public health guidelines. Second, relying on self-reported behaviors carries the risk of social desirability bias, as respondents may overstate their adherence to mask-wearing, handwashing, and other preventive measures. Although we complemented interviews with direct observation, the mere presence of data collectors may have temporarily improved performance (the Hawthorne effect), meaning that observed practices might not fully reflect routine behavior. Finally, by recruiting food stores and groceries, our sample may not represent larger or more varied food service settings, such as restaurants and industrial processors, where knowledge and practice patterns could differ substantially.

### Conclusion

In conclusion, less than half (191/384, 49.7%) of food handlers had sufficient knowledge about COVID-19. Our findings also showed that food handlers with previous food safety training practiced more than their counterparts did. As a result, researchers, food safety communicators, the media, and all other related sectors should work to educate food handlers to advance their health and food safety training. Food handler training courses should be prioritized by public health officials, especially during the COVID-19 pandemic.

## abbreviations

FAO: Food and Agriculture Organization
WHO: World Health Organization
